# Association of the nutritional risk index recorded prior to allogeneic hematopoietic cell transplantation with the clinical prognosis in children

**DOI:** 10.1002/jha2.1054

**Published:** 2024-12-18

**Authors:** Hitomi Yonesu, Satoru Hamada, Hideki Sakiyama, Shinobu Kiyuna, Tokiko Oshiro, Nobuyuki Hyakuna, Koichi Nakanishi

**Affiliations:** ^1^ Department of Medicine Faculty of Medicine University of Ryukyus Nishihara Japan; ^2^ Department of Pediatrics University of Ryukyus Hospital Nishihara Japan; ^3^ Okinawa Red Cross Blood Center Naha Japan; ^4^ Department of Pediatrics Graduate School of Medicine University of Ryukyus Nishihara Japan

**Keywords:** pediatrics, prognostic factor, stem cell transplantation

## Abstract

**Introduction:**

The nutritional risk index (NRI), calculated using serum albumin levels and body weight ratio is a known prognostic factor in adult hematopoietic cell transplantation (HCT). However, its usefulness in pediatric HCT settings remains unclear.

**Methods:**

In a retrospective study, we examined pre‐transplant NRI impact on outcomes in 82 pediatric patients undergoing allogeneic HCT.

**Results:**

The 2‐year non‐relapse mortality (NRM) rate was 7.94% (95% confidence interval [CI], 3.05%–19.8%) and 30.8% (95% CI, 16.7%–52.2%) in the high and low NRI groups, respectively (*p* = 0.0037).

**Conclusion:**

We found that poor nutritional status prior to pediatric HCT led to a worse prognosis, including increased NRM.

## 1 INTRODUCTION

Allogeneic hematopoietic cell transplantation (allo‐HCT) is a curative therapy for refractory hematological malignancies and bone marrow failure. However, mortality associated with transplant‐related complications is high at 10%; overcoming this problem is an important challenge for improving transplant outcomes [1]. Pre‐HCT conditioning, including high‐dose chemotherapy and/or total‐body irradiation, can cause digestive tract malabsorption and nutritional deficiencies. Malnutrition during the peri‐transplantation period results in negative allo‐HCT outcomes. Nutritional markers, such as body mass index (BMI), pre‐HCT weight loss, and serum albumin are associated with clinical outcomes, including overall survival (OS), non‐relapse mortality (NRM), and graft‐versus‐host disease (GVHD) [2, 3, 4]. In pediatric HCT patients, low serum albumin levels and BMI were associated with an increased acute GVHD and NRM risk [4]. Recently, the nutritional risk index (NRI)—a nutritional status indicator—has been reported as a prognostic factor in adult HCT [5]. The NRI—a simple index calculated using a combination of serum albumin levels and body weight (BW) ratio (current BW/ideal BW [IBW])—is used to evaluate nutritional status and predict clinical outcomes in the perioperative period and ICU management [6]. The usefulness and significance of the NRI in allo‐HCT in pediatric patients are unknown.

## 2 METHODS

We retrospectively analyzed the impact of the NRI before allo‐HCT on outcomes in pediatric settings. This study was approved by the University of Ryukyus Institutional Review Board and was conducted in accordance with the Helsinki Declaration. Pre‐HCT height, BW, and serum albumin levels were retrospectively collected from medical records.

The NRI was calculated as follows: (1.519 × serum albumin) + (41.7 × current BW/IBW). IBW was obtained based on the Japan Pediatric Endocrine Society criteria [7]. For boys, it was calculated as follows: < 6 years and 70–120 cm in height, IBW = 0.00206 × (height)^2^‐0.1166 × (height)+6.5273; ≥6 years and 101–140 cm in height, IBW = 0.0000303882 × (height)^3^‐0.00571495(height)^2^+0.508124 × (height)‐9.17791; ≥6 years and 140–149 cm in height, IBW = ‐0.000085013 × (height)^3^+0.0370692 × (height)^2^‐4.6558(height)+191.847; and ≥6 years and 149–184 cm in height, IBW = ‐0.000310205 × (height)^3^+0.151159 × (height)^2^‐23.6303 × (height)+1231.04. For girls, it was calculated as follows: < 6 years and height of 70–120 cm, IBW = 0.00249*(height)^2^‐0.1858 × (height)+9.0360; ≥6 years and height of 101–140 cm, IBW = 0.000127719*(height)^3^‐0.0414712 × (height)^2^+4.8575 × (height)‐184.492; ≥6 years and height of 140–149 cm, IBW = ‐0.00178766*(height)^3^+0.803922(height)^2^‐119.31*(height)+5885.03; and ≥6 years and height of 149–171 cm, IBW = 0.000956401*(height)^3^‐0.462755*(height)^2^+75.3058*(height)‐4068.31. Patients were assigned to the high (≥97.5) and low (<97.5) NRI groups according to the NRI score [5]. Body mass index (BMI) z‐score is based on BMI reference data of Japanese children [6 ]. Overweight and obese patients were defined as BMI z‐score ≧2.　Myeloablative conditioning was defined as total body irradiation > 8 Gy, melphalan > 140 mg/m^2^, or i.v. Busulfan ≥ 7.2 mg/kg. GVHD grade was assigned based on standard criteria [8]. The HCT‐specific comorbidity index (HCT‐CI) was scored [9]. The study examined OS, NRM, and relapse rates. OS was the time from transplantation to death from any cause, and NRM was death from other causes without relapse after transplantation. The relapse rate was the probability of hematologic malignancy relapse after transplantation. Dichotomous variables were compared using Fisher's exact test. OS probabilities were calculated using Kaplan‐Meier estimates. Cumulative incidence curves were used in a competing setting to estimate the probabilities of acute and chronic GVHD, NRM, and relapse using Gray's method. The regression analysis between NRI and BMI z‐score‐related indices was calculated with Spearman's correlation analysis. Statistical analyses were performed using the EZR software.

## 3 RESULTS

From January 2007 to September 2021, 82 patients underwent allo‐HCT at the Department of Pediatrics, University of Ryukyus. We excluded four overweight/obese patients. There were 52 and 26 patients in the high and low NRI groups, respectively.　Baseline characteristics are shown in Table 1.

**TABLE 1:** ALL indicates acute lymphoblastic leukemia; AML, acute myeloid leukemia; MDS, myelodysplastic syndrome; CML, chronic myeloid leukemia; *Others include three patients with congenital bone marrow failure, two patients with chronic active Epstein‐Barr virus infection, four patients with primary immunodeficiencies, two patients with aplastic anemia, one patient with congenital metabolic disorder, and one patient with primary hemophagocytic lymphohistiocytosis.

Patients' characteristic			
Factor	High NRI (*n* = 52)	Low NRI (*n* = 26)	*p*
Age, year	7.56 (5.59)	8.62 (5.93)	0.44
Sex			
Female	18 (34.6)	8 (29.6)	0.80
Male	34 (65.4)	19 (70.4)	
Disease			
ALL	16 (30.8)	6 (23.1)	0.25
AML	9 (17.3)	5 (19.2)	
ML	0 (0.0)	1 (3.8)	
Solid tumor	6 (11.5)	8 (30.8)	
MDS	4 (7.7)	3 (11.5)	
Aplastic anemia	6 (11.5)	1 (3.8)	
CML	3 (5.8)	0 (0.0)	
Others^*^	8 (15.4)	2 (7.7)	
Stem cell source			
BM	24 (46.2)	7 (26.9)	0.24
PBSC	10 (18.2)	8 (30.8)	
UCB	18 (34.6)	11 (42.3)	
HCT‐CI			
0	49 (92.7)	24 (92.3)	0.21
1–2	3 (7.3)	2 (7.7)	
≧3	0	0	
HLA			
Match	15 (27.3)	9 (34.6)	0.61
Mismatch	37 (72.7)	17 (65.4)	
Related donor			
Yes	25 (48.1)	14 (53.8)	0.24
No	27 (51.9)	12 (44.4)	
Myeloablative conditioning			
No	29 (55.8)	18 (69.2)	.33
Yes	23 (44.2)	8 (30.8)	
TBI			
No	24 (46.2)	10 (38.5)	0.43
Low	16 (30.8)	12 (46.2)	
High (≧8 Gy)	12 (17.3)	4 (15.4)	
GVHD prophylaxis			
CyA	13 (25.0)	9 (34.6)	0.51
PostCY/Tacrolimus/MMF	3 (5.8)	0 (0.0)	
Tacrolimus	36 (69.2)	17 (65.4)	

Abbreviations: CY, cyclophosphamide; MMF, mycophenolate mofetil.

**FIGURE 1:**
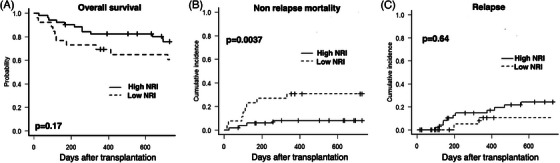
Two‐year probability of overall survival (OS) (A), cumulative incidence of non‐relapse mortality (NRM) (B), and cumulative incidences of relapse (C).

The 2‐year OS rates were 75.9% (95% confidence interval [CI], 61.4%–85.6%) and 60.6% (95% CI, 39%–76.6%) in the high and low NRI groups, respectively (*p* = 0.17) (Figure 1A). The 2‐year NRM rate was 7.94% (95% CI, 3.05%–19.8%) and 30.8% (95% CI, 16.7%–52.2%) in the high and low NRI groups, respectively (*p* = 0.001) (Figure 1B). The 2‐year cumulative incidence of relapse was 24.3% (95% CI, 14.2%–39.7%) and 10.5% (95% CI, 2.7%–35.9%) in the high and low NRI groups, respectively (*p* = 0.64) (Figure 1C).

**FIGURE 2:**
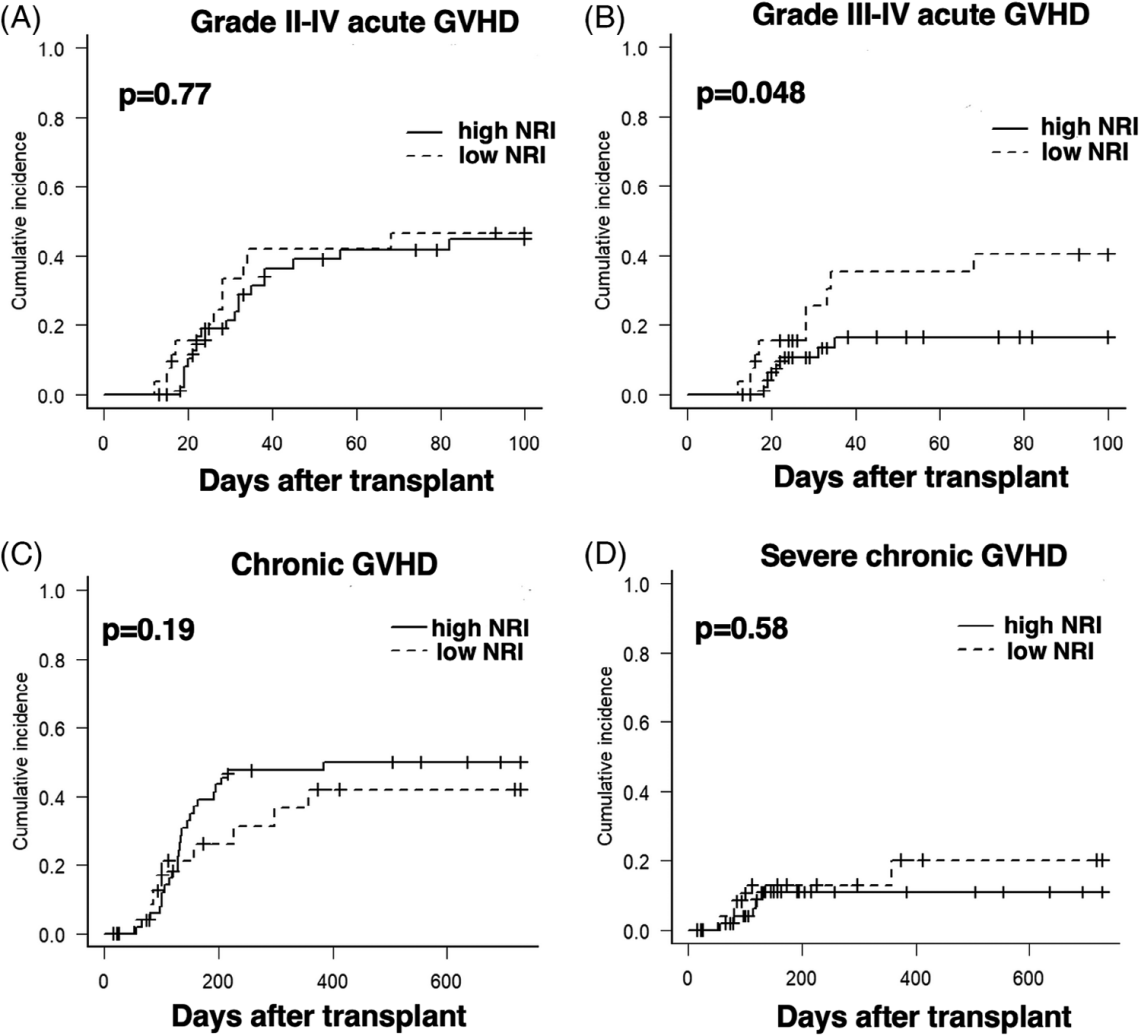
Cumulative incidences of grades II–IV aGVHD (A), cumulative incidences of grades III–IV aGVHD, cumulative incidences of cGVHD (C), and cumulative incidences of severe cGVHD.

The causes of NRM are summarized in Table S1. Thirteen patients died from causes other than relapse. In the low NRI group, infection was the most common NRM cause. The cumulative incidence of grades II–IV acute GVHD 100 days after HCT was 44.9% (95% CI, 31.1%–61.4%) and 46.7% (95% CI, 29.0%–68.4%) in the high and low NRI groups, respectively (*p* = 0.77) (Figure 2A). The cumulative incidence of grades III–IV acute GVHD was 16.5% (95% CI, 8.11%–31.9%) and 40.4% (95% CI, 23.2%–63.8%) in the high and low NRI groups, respectively (*p* = 0.048) (Figure 2B).

The cumulative incidence of chronic GVHD at 2 years was 52.7% (95% CI, 37.3%–65.2%) and 30.7% (95% CI, 14.8%–56.7%) in the high and low NRI groups, respectively (*p* = 0.19) (Figure 2C). The incidence of severe chronic GVHD at 2 years was 17.5% (95% CI, 7.44%–37.9%) and 15.7% (95% CI, 5.22%–41.8%) in the high and low NRI groups, respectively (*p* = 0.58) (Figure 2D).

## 4 DISCUSSION

The NRI is calculated using serum albumin as a biomarker and BW ratio as an anthropometric parameter. This index encompassing two nutritional status compositions reflects the nutritional condition of preoperative adult patients based on total parenteral nutrition. However, nutritional assessment using the NRI has not been reported in children. We evaluated the association between NRI and BMI z‐score using regression analysis. NRI was strongly correlated with BMI z‐score (R^2 ^= 0.60) (Figure S1). In pediatric heart transplantation setting, the BMI z‐score best predicted the post‐transplant metabolic syndrome in comparison to NRI [11]. Kerby et al. evaluated the malnutritional status in pediatric allo‐HCT by composing two variables called NUT 25, defined as albumin < 2.8 g/dL, weight loss ≧10% from baseline, and BMI < 25th percentile. Baseline NUT 25 was associated with an increased 100‐day mortality rate. Baseline BMI alone did not predict mortality; however, the addition of serum albumin level to define baseline nutritional risk led to better sensitivity in detecting baseline malnutrition and predicting 100‐day survival [4]. Moreover, in a pediatric HCT study, low pre‐HCT serum albumin levels were associated with an increased need for critical care interventions and 6‐month NRM caused by pulmonary toxicity and infection [10]. In our cohort, the most common cause of NRM was infection in the low NRI group.

Regarding GVHD, we found that low NRI was associated with an increased incidence of grades III–IV acute GVHD. In a previous study, weight loss and hypoalbuminemia in the early phase of transplantation predicted acute GVHD and early mortality [4]. Additionally, being underweight at the time of transplantation is associated with an increased acute GVHD risk in children [11]. Malnutrition alters the gut microbiota function and composition. Certain microbial taxa exert immunomodulatory effects on the recipient's adaptive immune system [12]. Therefore, a possible reason for acute GVHD in malnourished individuals may be gut dysbiosis, which produces inflammatory cytokines.

Our study has some limitations. This was a single‐center retrospective cohort study with heterogeneity in underlying diseases and transplant procedures among patients. Additionally, the small number of patients limits the interpretation of the results. Moreover, the cut‐off points for stratification by NRI should be validated by receiver operating curve analysis in larger cohorts of pediatric patients undergoing allo‐HCT.

In conclusion, high NRI indicates a good nutritional status, while low NRI signifies malnutrition and pre‐transplant malnutrition can predict poor transplant outcomes. Therefore, the need for pre‐transplant nutritional interventions must be addressed to decrease NRM.

## AUTHOR CONTRIBUTIONS

Hitomi Yonesu and Satoru Hamada conceived the study design. Hitomi Yonesu and Satoru Hamada collected data from electronic medical records. Hitomi Yonesu, Satoru Hamada, Hideki Sakiyama, Shinobu Kiyuna, Tokiko Oshiro, and Nobuyuki Hyakuna performed patient care. Hitomi Yonesu and Satoru Hamada wrote the first draft of the manuscript. Hitomi Yonesu, Satoru Hamada, Hideki Sakiyama, Shinobu Kiyuna, Tokiko Oshiro, Nobuyuki Hyakuna, and Koichi Nakanishi critically reviewed the first draft and approved the final draft of the manuscript.

## CONFLICT OF INTEREST STATEMENT

The authors declare no conflict of interest.

## ETHICS STATEMENT

The authors have confirmed ethical approval statement is not needed for this submission.

## PATIENT CONSENT STATEMENT

The authors have confirmed patient consent statement is not needed for this submission.

## CLINICAL TRIAL REGISTRATION

The authors have confirmed clinical trial registration is not needed for this submission.

## Supporting information



Supporting Information
FIGURE S1 NRI was correlated with serum albumin concentration (R^2 ^= 0.70) and %IBW (R^2 ^= 0.75).

Supporting Information
TABLE S1 Causes of NR.

## Data Availability

Data sharing is not applicable to this article as no new data were created or analyzed in this study.
