# Supplementary figures and images for: Association of the nutritional risk index recorded prior to allogeneic hematopoietic cell transplantation with the clinical prognosis in children
Source: EJHaem. 2024 Dec 18;6(1):e1054. doi: 10.1002/jha2.1054 (PMC11756969; doi:10.1002/jha2.1054)

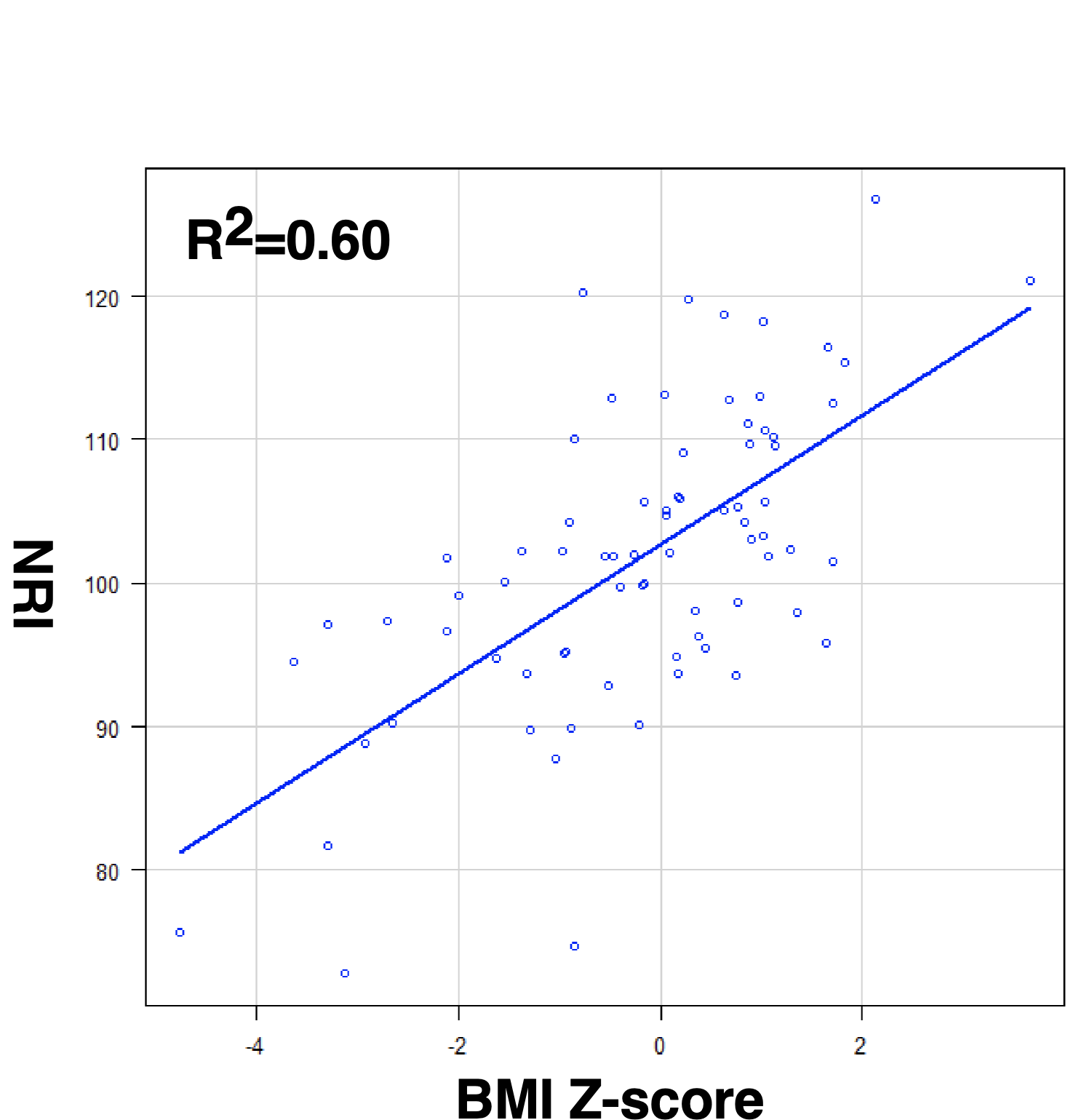

Supplement: Supplementary file 1 — Supporting Information FIGURE S1 NRI was correlated with serum albumin concentration (R2 = 0.70) and %IBW (R2 = 0.75). [file JHA2-6-e1054-s002.tif]
